# Irrigation in Endodontics: Polyhexanide Is a Promising Antibacterial Polymer in Root Canal Treatment

**DOI:** 10.3390/dj11030065

**Published:** 2023-03-01

**Authors:** Zurab Khabadze, Yulia Generalova, Alena Kulikova, Irina Podoprigora, Saida Abdulkerimova, Yusup Bakaev, Mariya Makeeva, Marina Dashtieva, Mariya Balashova, Fakhri Gadzhiev, Oleg Mordanov, Adam Umarov, Haddad Tarik, Andrei Zoryan, Amina Karnaeva, Yakup Rakhmanov

**Affiliations:** Department of Operative Dentistry, Institute of Medicine, Peoples’ Friendship University of Russia (RUDN University), Moscow 117198, Russia

**Keywords:** polyhexamethylene biguanide, PHMB, polyhexanide, apical periodontitis, *Enterococcus faecalis*

## Abstract

Background:chronic apical periodontitis is a common pathology in dentistry, especially in endodontics. It is necessary to systematize data concerning commonly used irrigation solutions. The development of new protocols for endodontic treatment is a very promising direction. The use of a polyhexanide-based antiseptic can positively affect the results of endodontic treatment. Methods: the review was carried out involving the search for English language research and meta-analyses in the Google Scholar and PubMed databases. Results: the number of literary sources that were identified during the literature review is 180. After excluding publications that did not match the search criteria, the total number of articles included in the systematic review was determined to be 68. Conclusions: polyhexanide is a promising solution for infected root canal irrigation. The antibacterial activity of this substance is suitable for the elimination of pathogens responsible for the appearance of apical periodontitis.

## 1. Introduction

Despite the development of the pharmacy industry and dental material science, the treatment and prevention of inflammatory diseases of the pulp and periodontium are quite complicated tasks.

The endodontist in most of his/her clinical cases has to deal with persistent infections in the root canals and their secondary infection, which leads to the presence of chronic infection in patients.

Pulpitis and apical periodontitis are inflammatory diseases of the pulp and periodontium, respectively, associated with a microbial factor. Microorganisms contaminating the root canal system from the carious cavity cause various pathophysiological processes in the tooth pulp. As a result of the lack of timely endodontic treatment, necrosis of the neurovascular bundle occurs. Microorganisms and their exo- and endotoxins begin to spread into the deep parts of the root canal and periapical tissues [1,2,3].

The microflora of primary and secondary endodontic infections is different, which complicates the selection of a root canal treatment protocol. Moreover, it should be taken into account that bacteria within the root canals are located not only in the form of plankton suspension, filling the main lumen of the canal, but also in the form of communities and consortia called biofilms. They are capable of penetrating into thin lateral branches, isthmuses, and even the outer surface of the root [4,5,6]. According to Siqueira J. and co-authors, up to 74% of teeth with progressive and sluggish apical periodontitis have multispecies biofilms [7].

These bacterial communities are definitely more resistant to antiseptic agents due to the presence of various factors (gene drift, antimicrobial withdrawal canals, and protective properties of the biofilm matrix), and there is increased interaction between aerobic and anaerobic bacteria [8,9,10]. Gram-negative bacteria of secondary colonization belonging to Fusobacterium, Porphyromonas, Prevotella, Dialister, Treponema, Tannerella, and Pyramidobacter, Gram-positive bacteria belonging to Parvimonas, Pseudoramibacter, Streptococcus, Enterococcus, Olsenella, Filifactor, Actinomyces, and Propionibacterium, and the fungi of the genus Candida (*Candida abicans*), according to the authors [1,2,3,4,5,6,7,11], persist in root canal systems with unsuccessful endodontic treatment, leading to the progression of apical periodontitis.

These microorganisms may show resistance to widely used irrigants in dentistry cationic agents (for example, chlorhexidine) due to the expression of resistance genes (qacE, qacEΔ1, and cepA for chlorhexidine), efflux pumps and changes in the hydrophobicity and permeability of cell membranes, the increased expression of membrane proteins OprF, LptD, and Tol-Pal, and the regulation of PagL, flagella proteins, chaperones, and proteins associated with energy metabolism [8].

Polyhexanide (international nonproprietary name) or PHMB is a polymer cationic antiseptic with a wide spectrum of antimicrobial, antifungal, and antiviral activity. The structure of PHMB is a base of 2–40 polymer biguanides and six methylene groups between them, combined with various mixes of aminoguanides or cyanoguanides as final groups [3].

The search for new antiseptic compositions for irrigation is a promising direction in dentistry. The use of polyhexanide (polyaminopropyl biguanide, PHMB) as a medicinal agent for washing the root canal system is an interesting question to discuss. In this review article we will consider information concerning the antimicrobial effect of polyhexanide on the microflora that causes inflammatory diseases of the tooth and near-root tissues. Special attention will be paid to the apical periodontium.

The systematization of available data on widely used irrigants and the search for new antiseptic compositions for irrigation are promising directions in dentistry. An interesting question is the use of polyhexanide (polyaminopropyl biguanide, PHMB) as a medicinal agent for the treatment of the root canal system. It has antimicrobial, antifungal, and antiviral activity. Information on the use of various irrigants in endodontic practice will be determined separately and in combination (pros and cons) with the spectrum of antimicrobial action of a polyhexanide.

## 2. Materials and Methods

The review was written during the search for the English language literature in the Google Scholar and PubMed databases.

Search terms and queries included the following: “microflora of apical periodontitis”, “secondary colonization of the canal”, “intra-root multispecies biofilms”, “irrigants in endodontics”, “antiseptic effects of irrigants”, “chemical interactions among antimicrobial solutions”, “chemical interactions among chelating agents”, “Polyhexanide”, “Polyhexanide AND dentistry”, “Polyhexanide AND endodontics”, “antibacterial effects AND *E. faecalis* AND Polyhexanide”, “antibacterial effects AND *C. albicans* AND Polyhexanide”, and “PHMB AND dentistry”.

The analysis included works that met the following selection criteria:

(1) Articles for the period from 2004 to 2023;
(2) An article describes the composition of the microflora that causes diseases of the pulp and apical periodontium;
(3) An article describes the use of various irrigants in endodontics (sodium hypochlorite, chlorhexidine, EDTA, and citric acid), as well as their interactions with each other;
(4) The research describes the outcomes of polyhexanide use in general medicine and dentistry;
(5) The article describes the spectrum of polyhexanide antimicrobial activity in the bacteria responsible for the occurrence of periodontitis.

**Figure 1:**
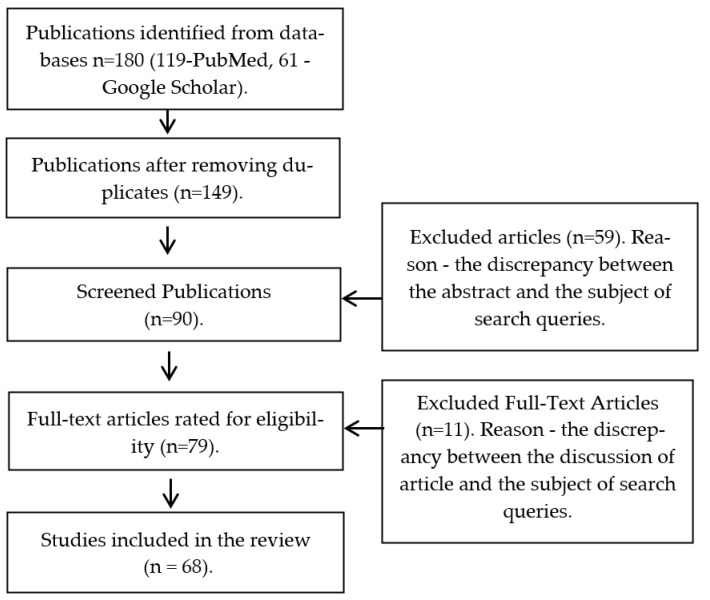
Article selection process.

The publications were selected and included in the analysis in several stages. In the first stage, repeated publications and works dated 2003 and earlier were excluded. Then, the title and brief content of the articles were analyzed for the presence of the key selection criteria, which were described above. After that, the content and full-text versions of the selected articles were considered (Figure 1).

## 3. Results

The number of literary sources that were selected during the review of the literature was 180, whereby 119 of which were in the PubMed electronic database and 61 were in Google Scholar. The total number of papers included in the systematic review became 68 after the deletion of repeated and unsuitable publications by the search criteria. Information on the microbial landscape of apical periodontitis, irrigants in endodontics, and the effect of polyhexanide on various types of bacteria was presented in full-text format in the publications.

## 4. Discussion

### 4.1. The Role of Root Canal Medical Treatment in the Pulp and Apical Periodontal Inflammatory Diseases. Properties of Irrigants

Inflammatory diseases of the pulp and periodontium make up a fairly large part of the total dental morbidity. A microbial factor is frequently linked to the onset and progression of pulpitis and periodontitis. Bacteria can enter the pulp space from the carious cavity through the dentine tubules (when the size of the dentine bridge is less than 0.5–0.2 mm), during dental treatment, through the destroyed dentoalveolar junction in marginal periodontitis [12,13,14,15,16,17,18,19,20,21,22,23,24].

High-quality endodontic treatment is the key to the success of complex therapy for pulpo-periodontal inflammatory diseases. The conservative therapy of the nosological units under discussion includes the mechanical treatment of the root canals with the help of manual and mechanical steel and nickel titanium instruments and drug therapy followed by three-dimensional obturation of the canal with gutta-percha pins and siler based on epoxy resins (other methods of filling the root canal system are also possible, provided that the root seal is hermetically sealed and there is no apical microleakage).

Proper preparation of the root canal during the mechanical stage of treatment creates conditions for irrigation. Medical treatment in endodontics is necessary since even the most flexible instruments are not able to fully contact all of the walls of the root canal, especially with those that are slit-shaped, C-shaped, dumbbell-shaped, and other irregular shapes. After preparation, pathogenic microorganisms remain on the walls of the channels, in isthmuses, ‘fins’, and other difficult-to-reach places, whereby the further reproduction of which can lead to the progression of the inflammatory process and a number of complications [13,14,15,16,17].

The issue of root canal irrigation has been widely discussed for a long time within the framework of endodontics. Researchers’ focus is aimed at creating a remedy with the best profile of antimicrobial activity and minimal cytotoxicity to periapical tissues and gingival fibroblasts. Practicing dentists and endodontists are interested in using a pharmaceutical drug that has a low reactivity with other irrigants (which makes it possible to use various active substances in combination without the risk of antagonistic interactions and the formation of toxic by-products).

The ideal root canal treatment method should have a broad spectrum of antimicrobial and antifungal activity, be substantial, have no irritating or cytotoxic effects on periapical tissues, cause no adverse reactions or interactions with other irrigation drugs, and not prevent root seal hardening. It must remain active in the presence of pus, blood, and other biological fluids. It should be noted that, in addition to the optimal antimicrobial spectrum of action, the ‘ideal irrigant’ should act on the organic components of the biofilms and the inorganic components of the lubricated layer for better disinfection and penetration of siler into dentine tubules [12,13,14,15,16,17,18,19,20,21,22,23,24,25].

There are currently no drugs on the market that have all of the aforementioned properties. Some data in the literature are based upon the use of proteolytic enzymes and synthetic antimicrobial drugs from the nitrofuran series as irrigants for root canals.

In practice, compounds based on iodine (iodinol), proteolytic enzymes, chloramine, and hydrogen peroxide are not recommended for use in endodontics. Iodinol can cause allergic reactions, irritation of periapical tissues, and staining of tooth tissue, and the use of hydrogen peroxide can lead to tissue emphysema. The action of proteolytic enzymes is unpredictable, and no pronounced antibacterial effect has been detected. Nitrofuran series preparations have also not been proven to be the drug of choice for root canal irrigation. The most widely used solutions were sodium hypochlorite (0.5–5.25%), chlorhexidine (0.02%, 2%), EDTA (17%), and citric acid [13,18].

Microbiologically justified and qualitatively performed root canal irrigation with antiseptic drugs creates the conditions for achieving the positive result of endodontic treatment. However, in practice, a dentist faces some difficulties. First, there is the spreading resistance of microorganisms to the medications used. Secondly, there is the negative impact of some drugs on the physico-mechanical qualities of intra-radicular dentin. For example, sodium hypochlorite in high concentrations (4–6%) with prolonged exposure is able to dissolve the organic basis of intra-root dentin, thus making the root brittle and susceptible to fractures. Furthermore, prolonged exposure to ethylenediaminetetraacetic acid (EDTA) leads to the depletion of dentin with minerals, which can subsequently lead to erosion. Third, the bacteria of endodontic infections exist not only in the form of planktonic suspensions, but also as part of biofilms, highly organized microbial communities united by an organic matrix mainly based on polysaccharides. Extracellular DNA (eDNA), enhancer RNA (eRNA), proteins, lipids, and other biomolecules are present in the matrix [12,13,14,15,16,17,18,19,20,21,22,23,24,25,26,27,28,29,30,31,32,33,34,35].

### 4.2. General Information about Irrigants

*Sodium hypochlorite*

This was first proposed for use in dentistry in 1936 [19]. It is a product with high rates of antimicrobial and proteolytic activity. The mechanism of action of NaOCl is based upon the following: upon contact with tissues, it decomposes to form hypochlorous acid and chlorine gas [16,20,21,22,23,24]. Chlorine has an antimicrobial effect and leads to the inactivation of bacterial enzymes due to the oxidation of SH-groups. The molecule of hypochlorous acid also determines the antimicrobial and proteolytic action.

Chlorine derived from NaOCl can take two chemical forms: hypochlorite (OCl-) or hypochlorous acid (HOCl). In dentistry, sodium hypochlorite is used with a pH of 11–12 [12,23,25], which defines it as a strong base. The higher the pH, the more of the substance is in the form of hypochlorite ions. Both forms have a pronounced antimicrobial and proteolytic effect, dissolving lipids and neutralizing amino acids.

An increase in temperature potentiates the antimicrobial effect of sodium hypochlorite, which determines the frequent use of a solution heated to 50–60 degrees in endodontics [12,26]. Additionally, sound activation (using the EndoActivator device) and ultrasonic activation using special nozzles are often used. The activation of the solution will contribute to its better penetration into dentine tubules.

The following fundamental points are pursued in the manufacturing of this solution: efficiency, safety, and stability. Sodium hypochlorite is a compound capable of self-degrading over time, and the decomposition process at room temperature is directly dependent on the concentration of the solution. A higher concentration leads to a less stable irrigant, and its shelf life decreases. Solutions of 3% NaOCl, widely produced on an industrial scale, are optimal based on the criteria specified above and do not require prolonged exposure in root canals to achieve a proteolytic and antimicrobial effect, as solutions with a lower concentration do. In clinical practice, the risk of a so-called hypochlorite accident is not forgotten when the solution penetrates beyond the root of the tooth as a result of exceeding the working length, the presence of lateral perforation, a “broken” apex, or the jamming of the irrigation needle. Moreover, the higher the concentration of NaOCl, the more pronounced the symptoms of parkane extrusion into the periapical tissues (pain, sudden appearance, and increase in swelling in the buccal, zygomatic, or infra-orbital zones, depending on the causal tooth, hematoma, and destructive effect on nerve trunks) [27,28,29,30,31,32,33,34,35,36].

It should be noted that sodium hypochlorite, because of the presence of proteolytic activity, can negatively affect the organic matrix of intracanal dentin. When depleted by organic compounds, the physico-mechanical parameters of the tooth root change—the tooth root becomes more brittle and less elastic.

*Chlorhexidine*

This is a synthetic cationic bisguanide with a broad spectrum of activity, including against pathogenic microorganisms that cause pulpo-periodontal complex infections, fungi, and lipophilic viruses. CHX is the gold standard of antiseptics in dentistry; it is used as a component of toothpastes, mouthwashes, and adhesive plates for the mucous membrane, and is also widely used in endodontics and periodontics. CHX has antimicrobial activity against many Gram-negative and Gram-positive bacteria. Insensitive bacteria can be multi-resistant *Staphylococcus aureus* (MRSA, VRSA), Pseudomonas spp., Proteus spp., acid-resistant forms of bacteria, and spores, whose outer cell layers form an impenetrable barrier to the penetration of CHX [28].

The principle of operation is based on the interaction of a positively charged chlorhexidine molecule with a negatively charged bacterial cell wall (phosphate groups of teichoic acid in Gram-positive bacteria and lipopolysaccharides in Gram-negative bacteria). According to the authors, in low concentrations (0.02–0.06%) it has a bacteriostatic effect, changing the osmotic balance of the cell, leading to the loss of up to 50% of potassium, phosphorus, and other ions, and in high concentrations (>0.12%) it is bactericidal, via cytolysis, leading to the complete release of the main intracellular components, including the number of nucleotides, to change the protein structure of the cell and the precipitation of cytoplasmic proteins. Low doses of CHX reduce the IL-1ß-induced secretion of PGE2, IL-6, IL-8, and MMP-1 via gingival fibroblasts [16,22,23,26,29].

Some authors have proposed using this substance as a final irrigant because it has substantiality, or the ability to be released for an extended period of time upon contact with the substrate (enamel, dentin, and glycoprotein groups). The release can last up to 12 weeks, according to some in vitro experiments [15,16,26,30].

Chlorhexidine does not exhibit proteolytic activity, and it does not act on the organic components of the biofilm. The death of microorganisms occurs only on the surface of the biofilm, since CHX is not able to penetrate deep into the polysaccharide matrix. Furthermore, having different ways of exchanging gene material, bacteria located deep in biofilms can acquire resistance to this antiseptic [31].

Particular attention should be paid to the ability of microorganisms in the deep layers of biofilms to use dead cells from the action of antiseptics located superficially as a substrate for nutrition, since cationic agents, including chlorhexidine, affect only the upper layers of biofilms.

Based on the above facts, chlorhexidine cannot be recommended for single use in endodontics, outside of combinations with other substances that have a solvent effect on biofilm polysaccharides, the lubricated layer, and necrotic pulp tissues.

*Ethylenediaminetetraacetic acid (EDTA) and citric acid*

EDTA is a complex molecule with a claw-like structure that binds and captures divalent and trivalent metal ions, such as calcium and aluminum, which form a stable ring structure, a chelator. It has a slightly alkaline or neutral reaction. EDTA affects the mineral components of dentin, and under certain conditions, it may have insignificant antimicrobial activity [32]. EDTA forms a stable complex with calcium. When all of the available ions are bound, EDTA’s reactivity is no longer possible [33].

In endodontics, it is used most frequently in a concentration of 15–17% to remove the lubricated layer formed after a root canal and to facilitate the sliding of instruments into the lumen of the root canal. It is often used in combination with sodium hypochlorite, as EDTA removes the lubricated layer that clogs the lumen of the dentine tubules, thus improving the further penetration of NaOCl into the dentine tubules. This achieves a more pronounced antibacterial effect and increases the stability of the root seal. The interaction between sodium hypochlorite and EDTA will be described in Section 3 of the article.

Citric acid is an organic acid used in endodontic practice in concentrations of 10–50% at pH 1–2 [32,33,34]. In addition to EDTA, citric acid is used to eliminate the lubricated layer formed on the surface of dentin by binding calcium ions. However, ethylenediamitetraacetic acid is still used more frequently at a concentration of 17% in routine therapeutic treatment.

None of the irrigation solutions currently available in dentistry can be considered to be ideal. The use of drug combinations in the correct sequence, considering their chemical interactions, contributes to the successful outcome of treatment. Solutions, even in small quantities, remain in the lumen of the root canal before applying the next irrigant and come into contact with each other, forming by-products. It is necessary to detail the chemical interactions of the substances used for root canal flushing to determine the optimal protocol for their use.

### 4.3. Interactions between Irrigants

Often, practitioners use more than one irrigant in a root canal drug treatment protocol. This is used to achieve better antiseptic treatment efficiency. All irrigation solutions and gels are different chemicals that can interact with each other. The results of such reactions can be both positive and negative outcomes that affect the quality and long-term indicators of the treatment of pulpitis and periodontitis. It is important to detail the nature of chemical interactions between irrigants in order to determine the most promising combinations. The main characteristics can be seen in Table 1.

### 4.4. Polyhexanide Is a Promising Irrigant. General Information and Application in Medicine

The mechanism of polyhexanide’s antimicrobial action as a cationic antiseptic is based on its connection with negatively charged phospholipids of the bacterial cell membrane by forming a polyhexanide–phospholipid complex, which leads to a violation of the integrity of the cytoplasmic membrane, a progressive decrease in the fluidity of the outer phospholipid layer, and the formation of hydrophilic sites in it. Polyhexanide is able to replace the magnesium and calcium ions that are part of the outer membrane of Gram-positive and Gram-negative microorganisms. Thus, this cationic antiseptic has a pronounced affinity for negatively charged prokaryotic cells. Additionally, PHMB targets are lipopolysaccharides in the outer membrane of Gram-negative bacteria, teichoic acids on the cell wall of Gram-positive bacteria, peptidoglycan components of the cell wall, and cytoplasmic membrane proteins [39,40].

The mechanisms described above negatively affect the osmoregulation and metabolic activity of the cytoplasmic membrane and enzymes, leading to the disruption of the vital activity of the microbial cell, an osmotic ‘explosion’, and its death. This agent does not act on the lipid membranes of eukaryotic cells or human DNA [3,7,41].

The ATP-dependent active penetration without disruption of the cell wall of PHMB is also possible, and then the inactivation of intracellular targets occurs. The fungicidal effect of polyhexanide is associated with the ability to perform electrostatic interactions with the cell wall, the accumulation in it, and the destruction of the nuclear membrane, leading to increased cell permeability, fragmentation, and DNA inactivation. A minimum inhibitory concentration of 1–2 µg/mL was shown for 50% of C. albicans cells and of 4 µg/mL for 90% [42].

Data concerning the ability of PHMB to interact with polysaccharides are available and can be useful in the inactivation of biofilms. The extracellular matrix of biofilms contains polysaccharide molecules that provide spatial stability and protection from antiseptic agents that do not affect the organic components of the films [37,41]. If PHMB is able to accumulate in the intracanal biofilm matrix, this fact makes it the drug of choice in the treatment of chronic apical periodontitis. Further studies are needed to establish the interaction of polyhexanide with the polysaccharide matrix of biofilms.

Because individual microorganisms react to the agent with varying degrees of sensitivity over time, a minimum contact time of the antiseptic with the substrate for 10–15 min is recommended for the optimal antimicrobial effect of polyhexanide.

Based on the data available in general medical practice, polyhexanide is not recommended for peritoneal lavage; the antiseptic washing of joint cavities; during operations on the central nervous system, elements of the peripheral nervous system, or the middle and inner ear; or during pregnancy (only if the risk to the mother and fetus is justified by achieving the targeted effects of the drug) [43]. It is worth noting that the shelf life of opened packages of commercially manufactured polyhexanide solutions, for example, Prontosan 0.1% and Lavacept 20% (B. Braun Melsungen AG, Melsungen, Germany), is not very long. After opening, Prontosan should be used within 6–8 weeks since there is a risk of contamination of the solution with spores. Lavasept, after preparing an antiseptic with the right concentration, must be disposed of after 24 h [44].

Pure solutions consisting only of the active substance PHMB are not represented in the pharmaceutical market. The composition of antiseptic compositions includes compounds such as 0.1% Undecylenamidopropyl Betaine (tenside) (in Prontosan (B. Braun Melsungen AG, Melsungen, Germany)) and macrogol 4000 (in Lavasept (B. Braun Melsungen AG, Melsungen, Germany), Lavasorb (Fresenius Kabi GmbH, Bad Homburg, Germany), Lavanid (Serag-Wiessner KG, Naila, Germany), and Serasept (Serag-Wiessner KG, Naila, Germany)). These substances can affect the surface tension of the solution and cytotoxicity, thereby improving the characteristics of the drug. According to some studies, polyhexanide does not have pronounced cytotoxic effects, its BI > 1, except for adverse effects on the chondrocytes of joints [45].

Undecylenamidopropyl Betaine (UB) is an antimicrobial surfactant that is commonly found in cosmetics. It is a derivative of undecylenic acid, a natural antifungal agent. In combination with polyhexanide, this substance is able to reduce the cytotoxicity of PHMB on fibroblasts responsible for the production of the collagen matrix. Undecylenamidopropyl betaine is presumably able to enhance the effect of polyhexanide on the fungi of the genus Candida. Further studies aimed at determining the synergistic and potentiating antimicrobial and fungicidal interactions of the components of Prontosan are promising in terms of solving the issue of the persistence of endodontic infection [46,47].

Works concerning the PHMB’s antibacterial activity relate mainly to the following sections of medicine: ophthalmology, there are studies in urology and gynecology, traumatology, the treatment of burns and wound infections, and long-term non-healing wounds, such as diabetic ulcers.

Polyhexanide (PHMB), triclosan, PVP-iodine, octenidine (OCT), and chlorhexidine (CHX) were tested for antimicrobial activity against *Staphylococcus aureus* (including methicillin-resistant *Staphylococcus aureus*), *Enterococcus faecalis* (including vancomycin-resistant Enterococcus), *Streptococcus pneumoniae*, *Escherichia coli*, *Pseudomonas aeruginosa*, *Clostridium perfringens*, *Haemophilus influenzae*, and *Candida albicans* by Koburger and co-authors with the use of microbiological microdilution and quantitative suspension tests. 

The selection of drugs was based on their ability to resist wound infection. For most of the tested microorganisms, with the exception of C. perfringens and H. influenzae, the minimum inhibitory concentration _24_ for polyhexanide was comparable to that of octenidine or was twice as much (from 0.5 to 4 mg/L for PHMB and from 1 to 8 for OCT), and was from two to eight times higher than the indicators for CHX (from 2 to 32 mg/L). The reduction in the quantitative parameters of the suspension test by 4.8–5.55 log (EN 1040) or 3.8–4.55 log (EN 1275) showed a clear dependence on the time of exposure of polyhexanide to microbiological samples. Thus, the best results for this drug were achieved with an exposure time of 360 min to 6 h (2.5 mg/L for *Staphylococcus aureus* and *Pseudomonas aeruginosa*); less outstanding results were demonstrated with contact for 1 min (500 and 250 mg/L for Pseudomonas aeruginosa and *Staphylococcus aureus*, respectively) [48]. However, all the same, these indicators do not go beyond the permissible values for use on the skin and mucous membranes. That is, it can be concluded that polyhexanide turned out to be more effective in the microdilution test than in the quantitative suspension test, which may indicate the sensitivity of the antiseptic to the conditions of the experiment. Additionally, it is worth conducting further studies on the activity of antiseptics, including polyhexanide, against pathogenic and opportunistic microbiota in the presence of organic pollutants such as blood and saliva.

Lenselink, E. and Andriessen, A. [9] used biocellulose wound dressings modified with polyhexanide to treat non-healing wounds, including diabetes ones. The study included patients with an average duration of wound infection from 6.8 to 216 weeks, the average size of wounds was 15.3 ± 14.5 cm^2^, and the topography of lesions was different. Within 24 weeks, 75% of the patients were cured; the rest had a significant decrease in the volume of the wound surface and the severity of the inflammatory process. The authors also noted the predominant formation of granulation tissue at the site of damage, which indicated a decrease in microbial load (biofilms) on the wound surface, the healing of skin pathology, and epithelialization.

In the study by Sripriya Ramasamy et al., the antibacterial activity of the polyhexanide-modified collagen matrix against bacteria causing wound infection was evaluated, including microorganisms such as *Staphylococcus aureus*, *Enterococcus faecalis*, and *Streptococcus mutans* (these pathogens are also responsible for the persistence of secondary endodontic infection occurrence). The inhibition zones for the studied microorganisms were 14 mm, 9 mm, and 12 mm, respectively, which indicate the proper activity of PHMB at a concentration of 0.01% [49]. The effect of polyhexanide on microorganisms causing the progression of wound infection was also investigated.

Polyhexanide was used as an instillation solution in the treatment of posttraumatic osteomyelitis of the pelvis and lower limb bones. The use of the negative pressure drainage method in conjunction with a PHMB-based antiseptic resulted in a reduction in the length of patients’ stays in the hospital and the frequency of osteomyelitis relapses from 59 to 10% [50]. The above data indicate the effectiveness of this antiseptic.

Polyhexanide is often used to irrigate urethral catheters in order to prevent infections of the genitourinary tract in urology. The authors have shown the potential of decolonizing catheters with clinical bacterial isolates [51,52,53]. In gynecology, the use of PHMB in the treatment of inflammatory and dysbiotic diseases of the vagina has been determined since it does not have an inhibitory effect on the growth of Lactobacillus spp. and does not have strong toxicity or allergenicity when used vaginally [54,55]. Polyhexanide is mentioned in the literature for the treatment of acanthamoebic keratitis [56,57,58,59].

Today, the use of polyhexanide in dentistry is not widely developed. This substance is used more often as a part of solutions for rinsing the oral cavity in order to prevent caries and diseases of the mucous membranes. We would also like to discuss some authors’ works on the microbiological foundations of polyhexanide use in dentistry, particularly endodontics. Table 2 contains brief excerpts and the main conclusions from several studies on the antimicrobial efficacy of polyhexanides in dentistry. More detailed information will be provided below.

Periodontitis is often a complication of caries. One of the ways to prevent periodontal diseases is to influence cariogenic microorganisms to eliminate them. Some caries pathogens are Streptococcus mutans, Lactobacillus acidophilus, Actinomyces viscous, and L. rhamnosus. Comparing the antibacterial activity of chlorhexidine bigluconate, polyhexanide, and octenidine, it was found that the low minimum inhibitory and bactericidal concentrations for the cariesogenic bacteria were inherent for octenidine and polyhexanide. Chlorhexidine required a higher concentration, except for *A. viscosus*, to achieve the same results [60].

The use of polyhexanide in endodontics is of particular interest. A limited number of works on the use of this substance as an irrigant or component of a filling material for root canals is presented in electronic databases. So, in two studies, the authors modified a zinc-eugenol oxide (ZOE)-based canal filling paste with the addition of PHMB to achieve more predictable antibacterial effects on resistant bacteria. The concentrations of paste ZOE + 0.05, 0.1, 0.2, 0.4, 0.6, and 0.8% PHMB were obtained. The authors tested their activity against *Enterococcus faecalis*. The researchers observed antimicrobial activity of the modified filling material at a level of 85% for 14 days, which is definitely longer than that of AH Plus; a fresh portion of this siler shows 60% activity for the elimination of *Enterococcus faecalis*, but the antimicrobial effect is practically absent after 24 h and 7 days [61]. Another study looked at the antimicrobial effects of modified eugenol zinc oxide paste with 0.8%, 1.0%, 1.2%, and 4% Polyhexanide on *E. faecalis*, *Candida albicans*, *E. coli*, and *Staphylococcus aureus*. The largest growth retardation zone after incubation at 37 °C was demonstrated for *E. coli*—46.24 mm—which the authors associated with the structure of the cell wall of Gram-negative bacteria. Smaller growth retardation zones were demonstrated for Gram-positive microorganisms—*E. Faecalis* and *S. Aureus*—at 41.52 mm and 42.18 mm, respectively [62]. Perhaps polyhexanide binds worse to the components of the Gram-positive bacteria cell wall, such as teichoic and lipoteichoic acids, which can explain the smaller diameter of the “clean zones”. This statement needs to be supported by further microbiological studies.

In addition, the authors have shown the antibacterial activity of glass ionomer cement with the addition of 0.2%, 0.4%, 0.6%, and 0.8% PHMB vs. S. Mutans [63].

As previously stated, one of the most commonly observed bacteria of secondary colonization is *Enterococcus faecalis*, which can penetrate dentine tubes up to depths of more than 500 m, sometimes to the dentine–cement interface. Furthermore, in infected root canals, C. albicans, a biofilm-forming yeast, is found in 20–25% of cases. Often, they get into the root canal system during the open method of periodontitis treatment. Candida has surface adhesion and retains its virulence factors for a sufficiently long period of time, leading to the penetration of fungal hyphae into deep areas of the dentine tubules [64].

In vitro, polyhexanide can inactivate growth and eliminate *Enterococcus faecalis* in 5 min. The authors experimentally examined the antimicrobial ability of a 0.2% solution of PHMB (Bigvasan IB10 (Arch Chemicals. Inc., Norwalk, UK)) and a 2.5% solution of sodium hypochlorite on removed, single-rooted, previously untreated teeth. In eight of eleven samples infected with *E. faecalis*, no bacterial growth was obtained after irrigation with 0.2% polyhexanide; in three samples, the contamination was 10^1^–10^3^ CFU/mL, determining the effectiveness of the solution. Similar results were obtained for sodium hypochlorite (lack of growth in three out of five samples). Similarly, in six samples infected with C. albicans, there was no further cell growth after antiseptic treatment with polyhexanide, and in five samples the contamination was 10^1^ CFU/mL. The use of 2.5% NaOCl did not achieve the complete elimination of Candida in any of the five samples tested [11]. In another study with a comparable design, similar results were obtained in the elimination of enterococcus and Candida from 10^3^–10^4^ to 0 CFU/mL when using polyhexanide [65].

However, in another ex vivo study on dentin samples of single-root extracted teeth, this antiseptic showed insufficient activity for *E. coli* and *E. faecalis* biofilms compared to chlorhexidine, povidone-iodine, and parachlorophenol [66]. The failure of PHMB use was due to its low 0.1% concentration, which is found in Prontosan (B. Braun Melsungen AG, Melsungen, Germany), since the experiment showed a minimum inhibitory concentration of polyhexanide of 0.2% [67]. More studies are needed not only on the nutrient media in Petri dishes but also on dentin samples from the root canals of extracted teeth to determine the clinically significant inhibitory concentrations of polyhexanide for periodontopathogens.

Polyhexanide can affect microorganisms for longer than chlorhexidine. On Day 30, PHMB showed a significantly greater decrease in CFU/mL than CHX for *Enterococcus faecalis*, *Staphylococcus aureus*, and *Candida albicans* [68].

**Table 2:** A review of studies on the microbiological activity of polyhexanides in relation to various bacteria that cause dental pathology.

Authors, Year of Publication	Title of the Article	Polyhexanide Form	Research Method	Tested Microorganisms	Main Conclusions
Esra Uzer Celik et al, 2016 [60]	Antimicrobial activity of different disinfectants against cariogenic microorganisms	PHMB 0.2% (2000 mg/L) solution	Spectrophotometry to detect MIC, microbial growth on TSA agar to determine the MBC	*S. mutans*, *L. acidophilus*, *A. viscosus*, *L. rhamnosus*	The lowest MIC and MBC against S. mutans (60 mg/L) were obtained from PHBM. The values of MIC and MBC for other microorganisms ranged from 20 to 120 mg/L
Wei Dong; Rui Chen; Yue-Ting Lin; Zi-Xiao Huang; Guang-Jie Bao and Xiang-Yi He, 2020 [60]	A novel zinc oxide eugenol modified by polyhexamethylene biguanide: Physical and antimicrobial properties	Zinc oxide eugenol sealers modified with different concentrations of PHMB (0.05, 0.1, 0.2, 0.4, 0.6, and 0.8%)	The microbiological direct contact test (DCT)	*Enterococcus faecalis*	Antimicrobial activity of the modified filling material at the level of 85% for 14 days
Nai, Z.; Han, Y.; Huang, Z.; Wang, J.; and He, X., 2020 [62]	Physical and biological properties of a novel root canal sealer modified by polyhexamethylene guanidine.	Zinc oxide eugenol fabricated with polyhexamethylene guanidine: 0.8, 1.0, 1.2, and 1.4%	Agar diffusion test and measuring the diameter of inhibition zone	*E. faecalis*, *C. albicans*, *E. coli*, *S. aureus*	The largest growth retardation zone after incubation at 37 C was demonstrated for *E. coli*—46.24 mm
Zhu, K.; Zheng, L.; Xing, J.; Chen, S.; Chen, R.; and Ren, L., 2022 [63]	Mechanical, antibacterial, biocompatible and microleakage evaluation of glass ionomer cement modified by nanohydroxyapatite/polyhexamethylene biguanide.	Glass ionomer cement modified with nanohydroxyapatite/polyhexamethylene biguanide (0.2, 0.4%)	Direct contact test, WST-8 assay	*S. mutans*	Modified GIC’s antibacterial rate vigorously raised to 88.5% compared with pure GIC. Addition of PHMB 0.4% increased the level of antimicrobial activity up to 96.5%
Mikic, I.M.; Andrasevic, A.T.; Prpic-Mehicic, G.; Matijevic, J.; Tadin, A.; and Simeon, P. P., 2013 [11]	The effect of polyhexamethylen biguanide on microorganisms in root canal	Bigvasan IB10 (Arch Chemicals. Inc., Norwalk, UK)—polyhexamethylen biguanide (PHMB) in concentration of 0.2%	Counting of colony-forming units during cultivation on blood agar after PHMB use and average colony-forming units per milliliter (CFU/mL) was calculated and log10-transformed	*E. faecalis*, *P. aeruginosa*, *C. albicans*	In eight of eleven samples infected with *E. faecalis*, no bacterial growth was obtained after irrigation with 0.2% polyhexanide, in three samples the contamination was 10^1^–10^3^ CFU/mL, in six samples infected with C. Albicans there was no further cell growth after treatment with Polyhexanide, and in five samples the contamination was 10^1^ CFU/mL
Mikić, I.M.; Cigić, L.; Kero, D.; Govorko, D.K.; Mehičić, G.P.; and Simeon, P., 2018 [65]	Antimicrobial effectiveness of polyhexamethylene biguanide on *Enterococcus faecalis*, *Staphylococcus epidermidis* and *Candida albicans*.	0.2% PHMB solution	Counting of colony-forming units during cultivation on blood agar after PHMB, then multiplied with the dilution factor and converted to CFU/mL	*E. faecalis*, *S. epidermidis* and *C. albicans*	The PHMB was significantly more efficient at reducing the number of all three tested microorganisms. The elimination of enterococcus and Candida was determined from 10^3^–10^4^ to 0 CFU/mL
Dammaschke, T.; Jung, N.; Harks, I.; and Schafer, E., 2013 [66]	The effect of different root canal medicaments on the elimination of Entero-coccus faecalis ex vivo.	Prontosan (B. Braun Meslungen AG, Melsungen, Germany)—0.1% PHMB	The CFU was counted at 8-fold magnification using a stereomicroscope, then multiplied with the dilution factor and converted to CFU/mL, subsequently	*E. faecalis*	Polyhexanide showed the lowest values of activity against *E. faecalis* compared with CHX 2% gel, 1% powder, Povidone-iodine, and ChKM

## 5. Conclusions

According to the literature review, polyhexanide is a promising solution for the irrigation of infected root canals in endodontics. The antibacterial activity of this substance is suitable for the elimination of pathogens responsible for apical periodontitis. Further microbiological studies are needed to determine the effect of polyhexanide on various periodontopathogens, not only *Enterococcus faecalis*, *Staphylococcus aureus*, and *Candida albicans*. It is also worth investigating the possibility of bacteria acquiring resistance to PHMB since its chemical structure is similar to chlorhexidine. For CHX, which is also a cationic antiseptic, there are various bacterial resistance mechanisms. It is necessary to exclude the possibility of cross-resistance in microorganisms with respect to the cationic agent polyhexanide.

## Figures and Tables

**Table 1:** Irrigant interactions.

	Sodium Hypo-chlorite	Chlorhexidine	EDTA	Citric Acid
Sodium hypochlorite		The formation of an insoluble red-brown or orange-brown precipitate-parachloraniline (PCA) [12,13,18,35,36,37,38].	The neutralization reaction occurs with the formation of HOCl, which then decomposes with the release of a small amount of free chlorine and oxygen, which reduces its antibacterial and proteolytic properties [20,36,37].	There is a decrease in the effectiveness of sodium hypochlorite, which is similar to that described for NaOCl and EDTA [36].
Chlorhexidine			A white-milk precipitate is formed—a “white cloud reaction”. The precipitate is made up of crystals of pure chlorhexidine, which dissolves with a pH change [32,36,37].	With citric acid chlorhexidine forms a milky solution, which is similar to that described for EDTA [36].

## Data Availability

Not applicable.
